# Cyclophosphamide Attenuates Fibrosis in Lupus Nephritis by Regulating Mesangial Cell Cycle Progression

**DOI:** 10.1155/2021/3803601

**Published:** 2021-11-15

**Authors:** Yuehong Ma, Ling Fang, Rui Zhang, Peng Zhao, Yafeng Li, Rongshan Li

**Affiliations:** ^1^Shanxi Key Laboratory of Kidney Disease, Department of Nephrology, Shanxi Provincial People's Hospital, Taiyuan, China; ^2^Shanxi Precision Medicine Center, Shanxi Provincial People's Hospital, Taiyuan, China; ^3^Shanxi Institute of Scientific and Technical Information, Taiyuan, China; ^4^Department of Dermatology, Shanxi Provincial People's Hospital, Taiyuan, China

## Abstract

**Objectives:**

Most patients with systemic lupus erythematosus (SLE) develop lupus nephritis (LN) with severe kidney manifestations. Renal fibrosis can be primarily attributed to overproliferation of mesangial cells (MCs), which are subject to drug treatment. Nevertheless, the detailed mechanisms remain elusive. We sought to identify the effect of cyclophosphamide (CTX), a drug commonly used for LN treatment, on MC proliferation and explore its underlying mechanisms. *Material/Methods*. Cell proliferation and fibrosis in mouse kidney tissues were determined by histopathology staining techniques. Flow cytometry was used for cell cycle analysis. Cell cycle regulators were examined *in vitro* following treatment of immortalized human MCs with platelet-derived growth factor subunit B (PDGF-B). Quantitative real-time PCR and western blot analyses were used to measure the mRNA and protein levels of candidate cell cycle regulators, respectively.

**Results:**

CTX inhibited cell overproliferation induced by platelet-derived growth factor subunit B *in vitro* and *in vivo*. CTX (40 mg/l) was sufficient to induce G0/G1 phase cell cycle arrest. CTX treatment downregulated many critical cell cycle regulators including cyclins and cyclin-dependent kinases but upregulated cyclin-dependent kinase inhibitors. Additionally, CTX-treated samples showed significantly reduced fibrosis, as indicated by lower expression of interleukin-1*β* and *α*-smooth muscle actin.

**Conclusion:**

CTX inhibits proliferation of MCs by modulating cell cycle regulator and therefore arresting them at G1 phase. CTX treatment significantly alleviates the severity of renal fibrosis. These findings provide novel insights into the mechanisms by which CTX affects LN.

## 1. Introduction

Systemic lupus erythematosus (SLE) is an autoimmune disease characterized by widespread inflammation in affected organs attacked by the immune system. Up to 70% SLE patients develop renal involvement, which are clinically diagnosed as lupus nephritis (LN). LN remains a leading cause of disability and death worldwide. Chronic nephritis, nephrotic syndrome, and even acute nephritis with alternating active and stable phases are commonly seen in LN patients [1, 2]. Additionally, 1 out 10 SLE patients ends up developing end-stage renal diseases [3, 4], and LN-related renal failure is the major cause of death among patients with SLE. While the clinical characteristics of LN are complex, glomerular injury represents a predominant symptom. Glomerular injuries are histologically classified based on glomerular immune complex deposition and mesangial cell (MC) overproliferation.

The three most abundant cell types in glomeruli are MCs, podocytes, and glomeruli epithelial cells, with MCs comprising approximately 1/3 of the total cell population. MC functions are vital in tissue homeostasis by providing structural support to the glomeruli and producing and maintaining the mesangial matrix. In addition, MCs can regulate the filtration surface area as a result of their contractility and can phagocytose apoptotic cells or immune components [5]. Finally, MCs are also involved in immune responses in the glomerulus [6, 7].

MC proliferation is triggered by renal cell injury or inflammation. Interfering with the cell cycle at any stage can lead to cell cycle arrest, ultimately rendering apoptosis [8]. Several studies have demonstrated that tacrolimus, cyclosporine A, and methylprednisone (MP) can inhibit the proliferation of MCs and are promising therapeutic agents in treating glomerular diseases [9–13]. Previous studies have shown that tacrolimus inhibits cell cycle progression by decreasing the percentage of cells in S phase and increasing the percentage of cells in G0/G1 phase [14]. Similarly, cyclosporine A also prevents human MCs from entering S phase in a dose-dependent manner. MCs exposed to low micromolar levels of cyclosporine A showed significantly increased apoptotic cells. In addition, treatment with MP inhibited the proliferation of human MCs (HMCs) in a time- and concentration-dependent manner. Similar to that with cyclosporine A, treatment with MP (1–10 mg/l) for 48 h also promoted HMC apoptosis [14].

Although cyclophosphamide (CTX) pulse is widely used to treat LN in clinical practice, how CTX affects cell cycle and renal fibrosis remains to be determined. 3H-thymidine incorporation assay showed similar patterns between control and CTX-treated groups (5 × 10^−5^ mol/L) , suggesting that CTX did not alter the mesangial cell cycle at low concentrations [15]. However, whether high dose of CTX influences the cell cycle remains unclear. Understanding the mechanisms by which CTX affects the cell cycle and fibrosis will be useful for disease monitoring and treating LN. We therefore initiated the present study to investigate how CTX influenced the cell cycle progression, apoptosis, and fibrosis of HMCs.

## 2. Materials and Methods

### 2.1. Cell Proliferation Assay

Cell Counting Kit-8 (CCK-8, Boster Biotechnology Co., Wuhan, China) was used to measure cell proliferation according to manufacturer's protocol. Briefly, HMCs were cultured in 96-well plates (1 × 10^5^ cells/well), followed by treatment with 20 ng/ml platelet-derived growth factor subunit B (PDGF-B) for 24 h with or without CTX (40 or 80 mg/l). The cells were then incubated in 10 *μ*l CCK-8 solution at 37°C for 2 h in the dark. The optical density (OD) was measured at 450 nm using a Biotek microplate reader (Agilent Technologies, Santa Clara, CA, USA). The cell viability was calculated using the following formula: cell survival (%) = (mean OD of treated cells/mean OD of control cells) × 100.

### 2.2. Animals

Six young (6–7-week-old) female C57BL/6J mice weighing 20 ± 1.5 g were purchased from the Laboratory Animal Center, Shanxi Provincial People's Hospital (Taiyuan, Shanxi). Female MRL/lpr LN mice were purchased from the Model Animal Institute of Nanjing University (induced from Jackson Laboratories, USA). The mice were housed under controlled environmental conditions (temperature 24 ± 2°C, 12 h light-dark cycle, and humidity 40%-70%), given free access to water, and fed a standard laboratory diet. The study was conducted in accordance with protocols approved by the Laboratory Animal Center, Shanxi Provincial People's Hospital. Twelve female MRL/lpr LN mice were randomly divided into 2 groups: LN group and LN+CTX group. Mice in the LN+CTX group were exposed to CTX by oral perfusion (20 mg/kg·d, 12 weeks), whereas the LN group received saline for 12 weeks. The animals were sacrificed 12 h after the last oral injection. Serum was collected by centrifuging at 5000 rpm for 15 min at 4°C and stored at –20°C for determining BUN and Scr levels. Renal tissues were obtained and kept in 10% neutral-buffered formalin and embedded in paraffin for histopathological analysis. Additional renal samples were immediately frozen in liquid nitrogen and stored at −80°C until analysis.

### 2.3. Flow Cytometry

Cell cycle arrest after cyclophosphamide (CTX) treatment was examined by flow cytometry. The human mesangial cells (HMCs) were harvested from T25 culture flasks (Nest Biotechnology, Wuxi, China) 24 h after treatment and fixed with 70% ethanol at 4°C for 24 h. After washing once with ice-cold phosphate-buffered saline, the cells were treated with ribonuclease (100 mg/ml) at 37°C for 30 min, followed by staining with propidium iodide (50 mg/ml) at 4°C for 30 min in the dark. The cells were analyzed by using an FC500 flow cytometer (Beckman Coulter, Beckman, Palo Alto, CA, USA) to determine the proportions of cells within the G1, S, and G2/M phases.

### 2.4. Quantitative Real-Time Polymerase Chain Reaction (qRT-PCR) Analysis

RNA was extracted from HMCs treated with 20 ng/ml PDGF-B for 24 h. The RNA concentration was measured using a Biotek microplate reader (Agilent Technologies, Santa Clara, CA, USA). 500 ng RNA was reverse-transcribed into cDNA using PrimeScript™ RT Master Mix (Takara, Shiga, Japan) according to manufacturer's protocol. qRT-PCR was performed with TB Green® Premix Ex Taq™ II (Tli RNaseH Plus) (Takara). Primer sequences used for qRT-PCR are as follows: CDK2 F-ATCTT TGCTG AGATG GTGAC TCG and CDK2 R-ACTTG GGGAA ACTTG GCTTG T, CDK4 F-TTGCG GCCTG TGTCT ATGGT and CDK4 R-CAAGG GAGAC CCTCA CGCC, cyclin E F-CCGGT ATATG GCGAC ACAAG and cyclin E R-CACAG AGATC CAACA GCTTC AT, cyclin D1 F-GATCA AGTGT GACCC GGACT and cyclin D1 R-CTTGG GGTCC ATGTT CTGCT, p21 F-ATGTG CACGG AAGGA CTTTG and p21 R-CGTTT GGAGT GGTAG AAATC TGG, and *β*-actin F-ACCTT CTACA ATGAG CTGCG and *β*-actin R-CCTGG ATAGC AACGT ACATG G. Relative gene expression was normalized to that of *β*-actin.

### 2.5. Western Blot Analysis

HMCs and kidney tissues were homogenized in lysis buffer (Boster Biotechnology Co., Wuhan, China). Proteins were separated by 10% SDS-PAGE and transferred onto a PVDF membrane (EMD Millipore, Billerica, MA, USA). Primary antibodies against cyclin D1, cyclin E, CDK2, CDK4, p21 (Santa Cruz Biotechnology, Dallas, TX, USA), IL-1*β* (ab234437; Abcam, Cambridge, UK), and *α*-smooth A (ab5694; Abcam) and corresponding secondary antibodies (Boster Biotechnology Co., Wuhan, China) were used. Membranes were developed and visualized using the Quantity One analysis system (Bio-Rad, Hercules, CA, USA).

### 2.6. Kidney Tissue Preparation for Pathology

Kidney tissues were collected from anesthetized mice after perfusion. To prepare sliced sections for histology, kidney tissue was fixed with 4% paraformaldehyde, embedded in paraffin, cut to 4 μm thickness, and stained with hematoxylin and eosin (HE) or periodic acid-Schiff (PAS) for morphological evaluation, PAS for mesangial expansion, and Masson for fibrosis. This study was approved by the local Ethics Committee of Shanxi Provincial People's Hospital, China (no.: 201987).

### 2.7. Data Analyses

Experimental data with three biological repeats were analyzed statistically by paired *t*-tests using the SPSS 23.0 software (SPSS, Inc., Chicago, IL, USA) and are presented as the means ± standard error of the mean.

## 3. Results

### 3.1. CTX Reduces Proteinuria and Improves Renal Function in MRL/lpr Mice

**Figure 1:**
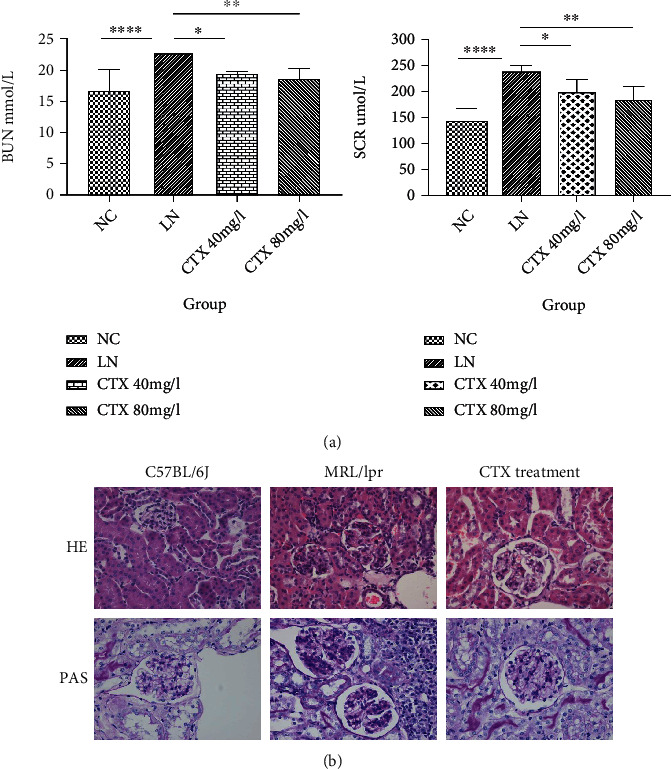
(a) Renal function in lupus nephritis after CTX treatment. CTX reduced the levels of blood urea nitrogen (BUN) and serum creatinine (SCR) of lupus nephritis (LN) mice. Data are presented as the means ± SEM (*n* = 6). ^∗^*p* < 0.05, ^∗∗^*p* < 0.001, and ^∗∗∗∗^*p* < 0.00001, lupus nephritis vs. control. (b) CTX 40 mg/l inhibits cell proliferation in glomeruli and necrosis of capillary loops. Glomerular pathology was detected by HE staining and PAS staining at week 17 (*n* = 6/group, 400x original magnification).

Treatment of MRL/lpr mice with 40 or 80 mg/l CTX ameliorated the symptoms of LN. Specifically, proteinuria, renal function abnormities, and kidney pathological lesions were reduced. The reduction in proteinuria was similar between the two dosage groups (Figure 1(a)).

### 3.2. CTX Inhibits Cellular Proliferation in Glomeruli *In Vivo*

To examine the cellular effect of CTX treatment on mice with LN, we have performed HE and PAS staining. Compared to the control mice, CTX-treated mice showed increased MC proliferation and accelerated progression of kidney fibrosis at week 12. We also examined both groups at week 17, and the difference was found to be more significant. Remarkably, mice treated with CTX for 12 weeks showed delayed disease progression compared to untreated mice. Pathological evaluation of MRL/lpr mice at week 17 revealed glomeruli with thickened pink capillary loops, which are typical of the so-called “wire loops” of LN. In mice treated with CTX, the surrounding renal tubules were unremarkable, and the opening of capillary loops was improved (Figure 1(b)).

### 3.3. MCs Treated with CTX Contribute to Decreased Inflammation and Fibrosis in LN

**Figure 2:**
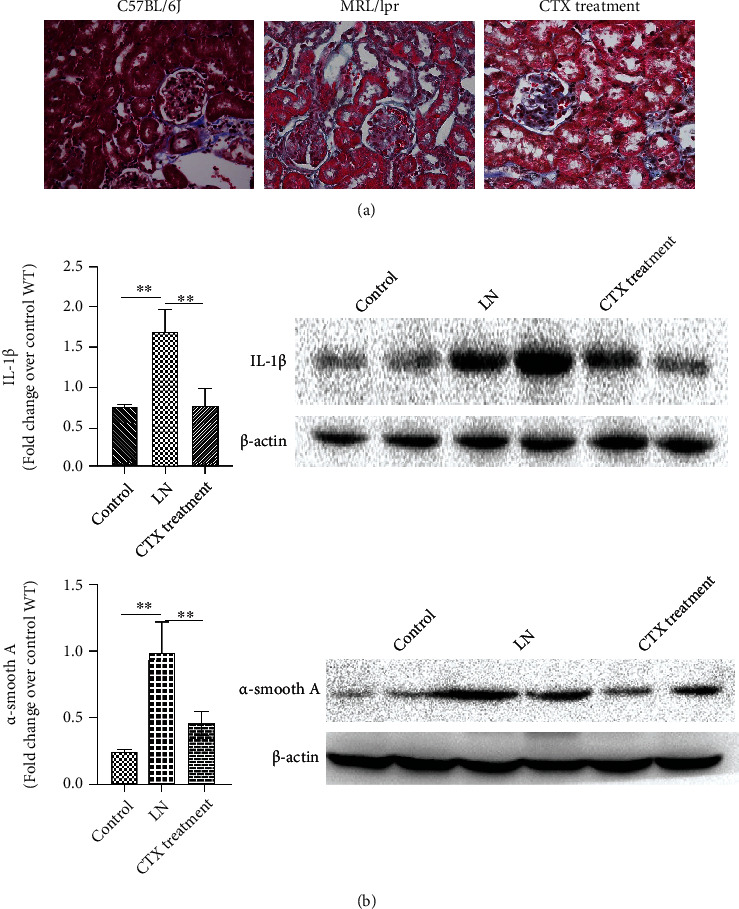
(a) CTX 40 mg/l delays the progress of kidney fibrosis. Glomerular pathology was detected by Masson staining at week 17 (*n* = 6/group, 400x original magnification). (b) Interleukin- (IL-) 1*β* and *α*-SMA in LN following CTX (40 mg/l) treatment. Multiplex analysis was used to determine the protein levels of IL-1*β* and *α*-SMA. The blots were representatives of independent biological triplicates and were analyzed by Friedman's test with Dunn's post hoc test, ^∗∗^*p* < 0.01.

Masson staining showed that CTX-treated mice delayed the progress of renal fibrosis, compared to the MRL/lpr LN mice (Figure 2(a)). And meanwhile, we investigated how CTX affects cellular inflammation and fibrosis. Interleukin- (IL-) 1*β* is a key cytokine mediating inflammatory responses while *α*-smooth muscle actin (*α*-SMA) is a marker for tissue fibrogenesis. IL-1*β* was expressed at relatively high levels in LN samples, indicating their inflammatory origins. However, as shown by the qRT-PCR and western blot results, IL-1*β* levels were significantly reduced following treatment with CTX, suggesting an ameliorated inflammatory response. *α*-SMA showed the same tendency following CTX treatment (Figure 2(b)). These results suggested that CTX could potentially mitigate both inflammation and fibrosis in LN patients.

### 3.4. CTX Reduces HMC Proliferation and Arrests the Cell Cycle in G0/G1 Phase

**Figure 3:**
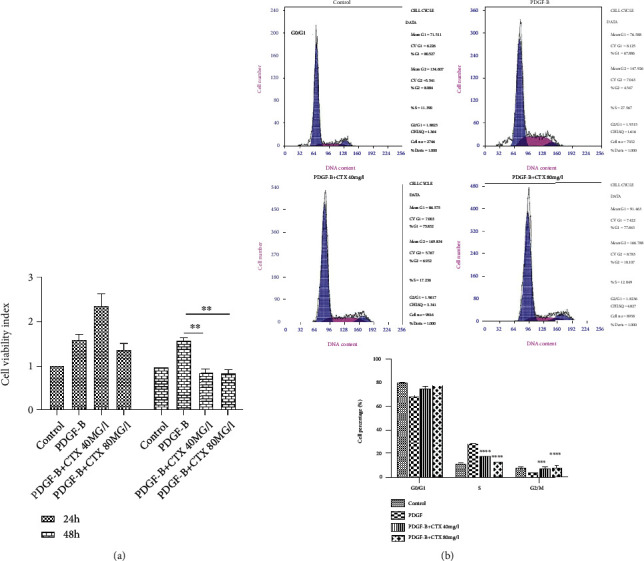
CTX reduces HMC proliferation by arresting the cell cycle in G1 phase. (a) CTX inhibited cell proliferation induced by PDGF-B at 48 h but not 24 h (*n* = 6/group). (b) Flow cytometry analysis showing cells in different phases of cell cycle. CTX arrested cells in G1 phase in a concentration-dependent manner; therefore, the percentage of cells in S phase was significantly lower (*n* = 6/group). Data are presented as the means ± SD, ^∗^*p* < 0.05, ^∗∗∗^*p* < 0.001, and ^∗∗∗∗^*p* < 0.0001.

We next assessed how CTX influences HMC proliferation *in vitro*. Cellular proliferation in CTX and PDGF-B-treated group was significantly reduced compared to that in cells treated by PDGF-B alone. We also found that the inhibitory effect of CTX depended not only on its dosage but also on its exposure time, although the difference was negligible at the high concentration. We found that neither 40 nor 80 mg/l CTX affected HMC proliferation following a 24-hour treatment. However, the inhibition rate of cell proliferation has the similar tendency with 24 h after treatment with 40 and 80 mg/l CTX for 48 h, but has significance in statistics (Figure 3(a)).

One plausible explanation of the reduced HMC proliferation upon CTX exposure was the shifted cell cycle. Therefore, we performed flow cytometry analysis to test this hypothesis. Consistent with our results of cellular proliferation, PDGF-B treatment increased the proportion of cells in S phase while decreasing the percentage of G1 cells. These results indicated that PDGF-B could shift cell cycle towards S phase (Figure 3(b)). In contrast, cells exposed to both PDGF-B and CTX were significantly enriched for the G1 phase population (*p* < 0.05), while deprived of the S phase population (*p* < 0.0001). These results suggested that CTX could override PDGF in halting the cells in G1 phase and blocking cell cycle.

### 3.5. Effects of CTX on Cyclins and Cyclin-Dependent Kinases

**Figure 4:**
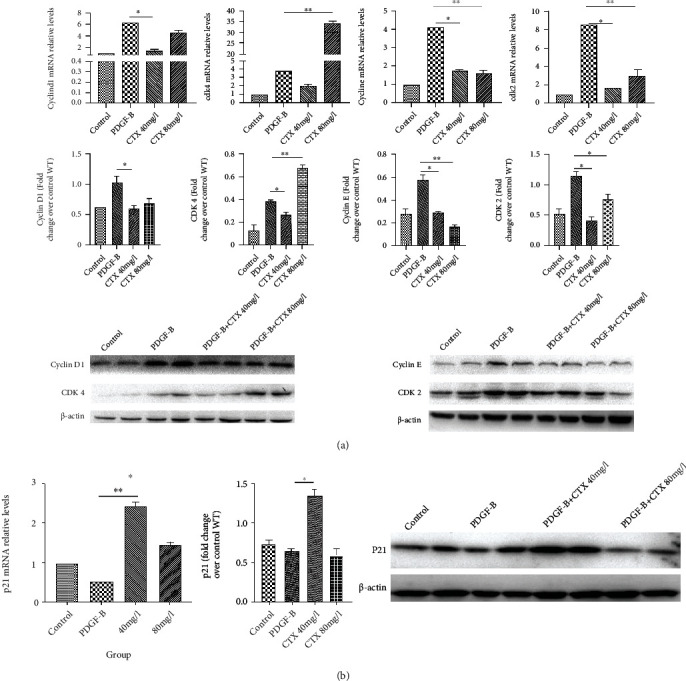
(a) Effect of CTX on candidate genes. HMCs were treated with 20 ng/ml PDGF-B for 48 h with or without CTX (40 or 80 mg/l). The expression levels of candidate genes were detected by western blotting and qRT-PCR. Blots were representatives of independent biological triplicates. ^∗^*p* < 0.05 and ^∗∗^*p* < 0.01. Data are presented as the means ± SD (*n* = 3). (b) Effect of CTX on cell cycle proteins. CTX (40 and 80 mg/l) upregulated p21 mRNA and protein in HMCs as demonstrated by qRT-PCR and western blotting assays, respectively. The western blots were representatives of independent biological triplicates. ^∗^*p* < 0.05 and ^∗∗^*p* < 0.01. Data are presented as the means ± SD (*n* = 3).

Cyclins and cyclin-dependent kinases (CDKs) are essential drivers of cell cycle events. To determine the mechanisms by which CTX affects HMC proliferation, we examined the expression of genes that are known to regulate G1 phase, such as cyclin D1, cyclin E, CDK2, and CDK4 (Figure 4). Both transcription and protein levels of the candidate factors were upregulated when cells were treated with 20 ng/l PDGF-B. Significantly, we found that treatment with CTX at 40 mg/l was sufficient to abolish the upregulation induced by PDGF-B. We also examined the effect of high dosage of CTX and found that 80 mg/l CTX inhibited the expression of all the proteins except for CDK4, which showed higher levels than that in PDGF-B-treated cells (Figure 4(a)).

### 3.6. CTX Reverses the PDGF-B-Induced Decrease in p21 Level

To gain further insights into how CTX regulates cell cycle components, we focused on p21, a cyclin-dependent kinase inhibitor, in quiescent and proliferating HMCs. In quiescent HMCs, both the mRNA and protein levels of p21 were detectable. However, the expression of p21 was decreased in cells incubated with PDGF-B. Significantly, this effect was reversed by 40 mg/l CTX treatment. Interestingly, treatment with 80 mg/l CTX only decreased p21 mRNA expression but not protein expression (Figure 4(b)). Altogether, these results suggested that CTX can reverse the downregulation of cyclin-dependent kinase inhibitor induced by PDGF-B exposure.

## 4. Discussion

Many SLE patients develop LN that is associated with poor prognosis and increased morbidity and mortality [16]. One shared phenotype of many glomerular diseases, including LN, is MC overproliferation [12]. MCs are involved in many biological processes in the renal glomerulus, such as secreting cell matrix, producing cytokines, and supporting glomerular capillary plexus, phagocytosis, and clearance of macromolecular substances, as well as contraction of smooth muscle cells. Once activated by inflammatory stimuli, MCs can also interact with migrating and infiltrating inflammatory cells, which in turn amplifies local inflammation responses, fibrosis, and the development of glomerulosclerosis [17, 18].

Similar to tacrolimus and cyclosporine A, MP is known to interfere with cell cycles by inducing G1-phase arrest and preventing cells from entering mitosis [14]. Consistent with this result, another study showed that glucocorticoids could decrease S/G2/M-phase populations in HEK293 cells by suppressing NF-*κ*B activities [19]. Mounting evidence has shown the antiproliferative effects of glucocorticoids across numerous cell types [20–24], and MP is likely to be effective on CDK inhibitors such as p21/Cip1 and p57/Kip2 [25, 26]. Alternatively, MP may also suppress the expression of c-myc or cyclins, which can stimulate cell cycle progression [20].

To dive into the prime feature of LN, we first analyzed the effects of CTX treatment on cell proliferation *in vitro*. Cell cycle progression is tightly regulated and coordinated by growth factors, oncogenic stimuli, and regulatory components such as cyclin/CDK complexes [27, 28]. Our data showed that the cell cycle was shifted by treating MCs with 40 mg/l or 80 mg/l CTX. As our results indicated that CTX mainly affected G0/G1 phase, we focused on regulators of these stages. Cyclin D and cyclin E, together with CDK2 and CDK4, facilitate the transition from G1 to G2 phases, whereas p21 has a strong inhibitory effect. Our results showed that PDGF-B could upregulate cyclin D, cyclin E, CDK2, and CDK4, while the expression of p21 was markedly reduced. Interestingly, in proliferating MCs, CTX downregulated the expression of cyclin D, cyclin E, CDK2, and CDK4 and upregulated p21 expression. These results suggested an antiproliferative effect of CTX in MCs.

An *in vitro* model of LN suggested that MCs could contribute to renal inflammation by secreting proinflammatory cytokines to local niches [7]. Studies based on human and animal models also showed that renal tubular epithelial-myofibroblast phenotypic transformation is critical in the pathogenesis of renal diseases associated with renal interstitial fibrosis, where *α*-SMA is an important marker of this transformation [29]. Additionally, fibrosis and key inflammatory factors, such as IL-1*β*, appear to be closely related [30].

Our study indicated that CTX could benefit renal fibrosis patients by targeting key cell cycle regulators. We found that expression of the fibrotic protein *α*-SMA and inflammatory factor IL-1*β* was markedly reduced in the LN mouse model after treatment with CTX, which delayed the progression of renal fibrosis and inflammation. Although we did not observe the same impact of CTX on HMCs, this discrepancy can be explained by the different concentrations of CTX administered *in vitro* and *in vivo*. While CTX (5 × 10^−5^ mol/L) did not alter the mesangial cell cycle [15], these data reveal a strong correlation between cell cycle, inflammation, and fibrosis. As an important hub of LN-related phenotypes including cell proliferation and immune cell activation, MC remains to be a promising therapeutic target. Our work elucidated detailed mechanisms by which CTX attenuates MC overproliferation and renal fibrosis and therefore laid a foundation for CTX therapy.

## 5. Conclusion

**Figure 5:**
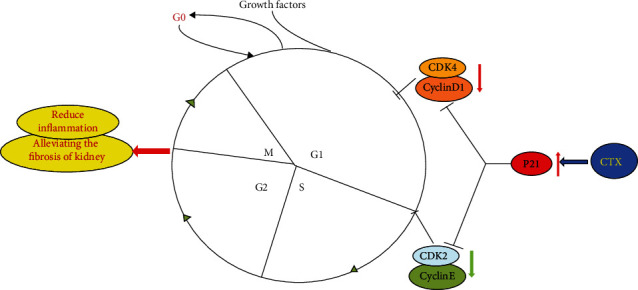
Proposed mechanisms of effects of CTX on mesangial cell proliferation.

In conclusion, a high dose of CTX inhibited the proliferation of HMCs and induced HMC apoptosis. Moreover, CTX dramatically reduced the expression of IL-1*β* and *α*-SMA, which are involved in the inflammatory response and fibrosis within the glomerulus. Therefore, we proposed that CTX might regulate cell proliferation by controlling the production of intracellular inflammatory fibrosis mediators and the cell cycle regulators. Overall, these data laid the foundation of CTX shock therapy for LN (Figure 5).

## Data Availability

The data used to support the findings of this study are available from the first author upon request.
